# Advancements in the Underlying Pathogenesis of Schizophrenia: Implications of DNA Methylation in Glial Cells

**DOI:** 10.3389/fncel.2015.00451

**Published:** 2015-12-02

**Authors:** Xing-Shu Chen, Nanxin Huang, Namaka Michael, Lan Xiao

**Affiliations:** ^1^Department of Histology and Embryology, Chongqing Key Laboratory of Neurobiology, Third Military Medical UniversityChongqing, China; ^2^College of Pharmacy and Medicine, Joint Laboratory of Biological Psychiatry Between Shantou University Medical College and the College of Medicine, University of ManitobaWinnipeg, MB, Canada

**Keywords:** DNA methylation, glial genesis, oligodendrocyte, astrocyte, schizophrenia, mental illness

## Abstract

Schizophrenia (SZ) is a chronic and severe mental illness for which currently there is no cure. At present, the exact molecular mechanism involved in the underlying pathogenesis of SZ is unknown. The disease is thought to be caused by a combination of genetic, biological, psychological, and environmental factors. Recent studies have shown that epigenetic regulation is involved in SZ pathology. Specifically, DNA methylation, one of the earliest found epigenetic modifications, has been extensively linked to modulation of neuronal function, leading to psychiatric disorders such as SZ. However, increasing evidence indicates that glial cells, especially dysfunctional oligodendrocytes undergo DNA methylation changes that contribute to the pathogenesis of SZ. This review primarily focuses on DNA methylation involved in glial dysfunctions in SZ. Clarifying this mechanism may lead to the development of new therapeutic interventional strategies for the treatment of SZ and other illnesses by correcting abnormal methylation in glial cells.

## Introduction

Schizophrenia (SZ) is a chronic and severe mental illness that affects approximately 1% of the population (Roussos and Haroutunian, 2014). This mental illness is characterized by clinical deficits featured by socially inappropriate behaviors such as paranoia, hallucinations, and inappropriate emotional responses. Although the pathogenesis of SZ is unknown, it has been commonly accepted to be caused by a combination of genetic, biological, psychological, and environmental factors (reviewed in Akbarian, 2014; Shorter and Miller, 2015).

In the past decade, several hypotheses concerning the pathogenesis of SZ have been raised up and tested. Among them, dysfunction of neurotransmitters, such as dopamine, gamma-aminobutyric acid (GABA), glutamate, as well as 5–hydroxytryptamine (5-HT) have been implicated as primary etiologies of SZ. In general, most studies have focused on the dysfunction of neurons in different regions of gray matter that include the ventral tegmental portion, the limbic system and the prefrontal cortex (Plitman et al., 2014; Taylor and Tso, 2015).

However, recent evidence has demonstrated the involvement of glial changes in the underlying pathogenesis of SZ. For example, in SZ, ultra-structural signs of oligodendrocyte deficiency have been implicated in myelinated fiber damage in gray matter (Barley et al., 2009; Williams et al., 2013b; Hercher et al., 2014). Moreover, increasing evidence from neural imaging, genetic analysis, post-mortem morphological, molecular biological and pharmacological studies revealed a wide range of white matter abnormalities such as dysfunctional oligodendrocytes with associated myelin deficits in the patients with SZ (Hof et al., 2002; Flynn et al., 2003; Voineskos et al., 2013; reviewed in Nave and Ehrenreich, 2014). Evidence has also shown that the abnormal activation of astrocytes or microglia may also be implicated in SZ (Katsel et al., 2011a; Frick et al., 2013). As such, glial cell dysfunction seems to be a primary deficit in SZ, which can alter the synaptic function and/or circuitry and cause neuronal deficits (Stewart and Davis, 2004; Segal et al., 2007; Barley et al., 2009; Williams et al., 2013a). Therefore, glial cells may function as key players that drive the underlying pathology of SZ (recently reviewed in Bernstein et al., 2015; Wang et al., 2015). The advanced understanding of the mechanisms that regulate glial function may provide a new insight into the development of therapeutic intervention strategies for SZ or related mental illnesses.

Most glial cells (i.e., astrocytes and oligodendrocytes) are differentiated from the neural stem cells (NSCs). After specification, glial progenitor cells/oligodendrocytes precursor cells (OPCs) can further differentiate into oligodendroglial lineage cells or astrocytes, and the processes are controlled by specific transcriptional programs (de Castro et al., 2013). Recently, studies have implicated specific epigenetic changes to SZ pathology (reviewed in Akbarian, 2014; Shorter and Miller, 2015). Epigenetic does not alter gene sequences but can directly influence gene expression (reviewed in Chen and Riggs, 2011; Smith and Meissner, 2013). For example, DNA methylation, one of the earliest types of epigenetic modifications, has been extensively linked to SZ (reviewed in Grayson and Guidotti, 2013). It has been shown that DNA methylation was correlated with oligodendrocyte dysfunction and/or myelin deficits in the brain of patients with SZ (Iwamoto et al., 2005, 2006; Wockner et al., 2014). Moreover, antipsychotic drugs have been shown to be able to alter the pathological changes associated with DNA methylation or demethylation and are thereby thought to be beneficial in the treatment of SZ (reviewed in Guidotti and Grayson, 2014). In this comprehensive review, we primarily focus on the role of DNA methylation in glial cells’ generation and aberrant methylation involved in glial dysfunction leading to SZ and other related illnesses.

## Glial Abnormalities in SZ

Glial cells mainly include astrocytes, oligodendrocytes, microglia, ependymal cells and retinal Müller cells in the central nervous system (CNS). In addition, the term glial cells also extend to Schwann cells and satellite cells in the peripheral nervous system (PNS). Besides basic functions like nutritional support or neuronal protection, glial cells have other integral roles including governing myelination, synapse formation and immunological reactions (Namihira and Nakashima, 2013). Increased evidence shows that glial cells, especially oligodendrocyte dysfunction with corresponding myelin deficits occur in the white matter or gray matter of SZ (reviewed in Bernstein et al., 2015; Miyata et al., 2015). Additional abnormalities have also been noted in astrocytes and microglia (Katsel et al., 2011a; Chew et al., 2013; Frick et al., 2013).

Oligodendroglial cells are generated from neural progenitor cells (NPCs) in the gliogenic phase of gestation. After specification, OPCs continued to differentiate into immature oligodendrocytes and finally the mature oligodendrocytes (de Castro et al., 2013). There are different oligodendrocyte markers identifying different stages of oligodendrocytes, including platelet derived growth factor receptor alpha (PDGFRα) for OPCs, O4 for immature oligodendrocytes, myelin associated glycoprotein (MAG), proteolipid protein (PLP), and myelin basic protein (MBP) for the mature oligodendrocytes (de Castro et al., 2013). Abnormal expression of these markers along with some oligodendrocyte related transcription factors have been found in SZ.

For example, PLP1 was shown to be down-regulated in SZ. It is believed that genetic polymorphisms of PLP1 in males are likely to cause an increased susceptibility to SZ (Qin et al., 2005). Similarly, a genetic association of the 2,3-cyclicnucleotide-phosphodiesterase (CNP) and MAG genes were also found in SZ (Wan et al., 2005; Yang et al., 2005). The CNP risk polymorphism was associated with down-regulation of gene expression in SZ (Peirce et al., 2006). Other studies have shown that although the expression of MAG, CNP and oligodendrocyte transcriptional factor 2 (Olig2) in the gray or white matter did not differ between SZ patients and normal controls, myelin-associated oligodendrocytic basic protein (MOBP) mRNA levels were increased in the white matter of patients with SZ (Usui et al., 2006). The myelin oligodendrocyte glycoprotein (MOG) gene is considered to be a key biological target for SZ due to its association with white matter abnormalities and its importance in mediating the complement cascade (Zai et al., 2005). Some oligodendrocyte related transcriptional factors, such as Olig2, a basic helix-loop-helix (bHLH) oligodendrocyte transcription factor, together with Olig1, are required for normal oligodendrocyte development and functioning. Genetic association analysis showed that variations in oligodendrocyte transcription factor Olig2 together with Olig1 result in abnormal oligodendrocyte development and functioning, thereby enhancing susceptibility to SZ (Georgieva et al., 2006; Prata et al., 2013). Another target factor called sex-determining region Y-box contains gene 10 (Sox10), has also been shown to be responsible for the terminal differentiation of oligodendrocytes by combining with Olig1/2 (Li et al., 2007; Küspert et al., 2011). The abnormal expression of Sox10 has also been found to be associated with SZ (Iwamoto et al., 2005).

Moreover, Disrupted-in-schizophrenia 1 (DISC1) has also been cited as a strong candidate target gene for SZ. Rationale involving its implication in SZ revolves around the ability of DISC1 to negatively regulate the differentiation of oligodendrocytes (Katsel et al., 2011b; Boyd et al., 2015). The neuregulin 1 (NRG1)/ErbB signaling pathway is one of the important pathways responsible for the regulation of myelination (Tao et al., 2009). Henceforth, it was also considered as a key target for intervention to assist the treatment for SZ (Alaerts et al., 2009). Moreover, recent evidence suggests a key role of astrocytes in SZ due to their ability to interact with neurons, and function in the maintenance of glutamate homeostasis and recycling. The expression of astrocytic genes such as S100 beta (S100β), diodinase type II, aquaporin-4, glutaminase, excitatory amino-acid transporter 2 (EAAT2) and thrombospondin are significantly reduced in the deep layers of the anterior cingulate gyrus in patients with SZ (Katsel et al., 2011a). In addition, post-mortem and imaging studies have also suggested a critical role for microglial activation in the underlying pathogenesis of SZ patients (Frick et al., 2013). As such, these recent findings suggest that glial dysfunction may be involved in the underlying pathogenesis of SZ. However, the exact molecular mechanisms underlying the pathological process are currently unknown.

### DNA Methylation

**Figure 1:**
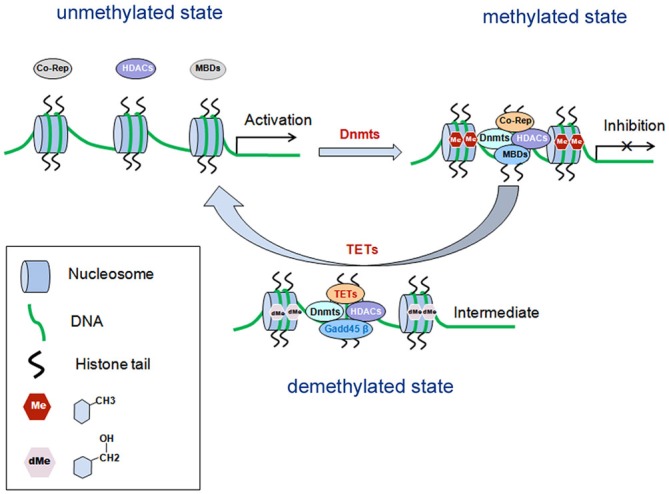
**Schematic diagram: DNA methylation in gene transcriptional regulation.** DNA methylation is mediated by Dnmts, which is recruited by MBDs and form transcription repressor complexes together with co-repressors (co-rep) and HDACs, and induces transcriptional inhibition. Demethylation is mediated by TETs, which can catalyze oxidation of 5mC to 5-hydroxymethylcytosine (5hmC), and finally leads to disassembly of the repressor complexes and gene transcriptional activation. MBDs, methyl-binding domain proteins; Dnmts, DNA methyltransferases; HDACs, histone deacetylase; Co-Rep, co-repressors; TETs, the ten-eleven translocases; Gadd45β, growth arrest and DNA damage 45-beta.

DNA methylation is one of the earliest found epigenetic modifications thought to drive the pathology of various psychiatric diseases such as SZ (Grayson and Guidotti, 2013; Numata et al., 2014). It is mediated by DNA methytransferases (Dnmts), which can transfer a methyl group (CH3) from S-adenosyl-L-methionine (SAM) to the fifth carbon position of cytosine. The methylated cytosine at fifth carbon position is named 5-methylcytosine (5mC). During the methylation process, Dnmts transfer the methyl group from SAM to cytosine residues, generating S-adeno-sylhomocysteine (SAH; reviewed in Smith and Meissner, 2013). In general, the methyl-binding domain proteins (MBDs) recruit histone deacetylase (HDACs) and co-repressors (co-rep) by Dnmts to form transcription repressor complexes. By forming the complexes, they prevent transcription factors from binding with their specific DNA sequences, causing silence or inhibition of gene expression (reviewed in Smith and Meissner, 2013; Figure 1). There are six members belonging to the MBD family, including MBD1–4, MeCP2 (methyl-CpG-binding protein 2) and Kaiso. At present, MeCP2 have two biologically active isoforms called MeCP2E1 and MeCP2E2 (Olson et al., 2014). Dnmts isoforms include Dnmt1, which maintains the methylation on the genes, and Dnmt3a and Dnmt3b for* de novo* establishment of DNA methylation (reviewed in Houston et al., 2013; Smith and Meissner, 2013). Cytosine methylation of promoter regions usually represses gene transcription (Figure 1). Conversely, demethylation is the process which removes a methyl group (CH3) from 5mC, finally a hydrogen atom is added to the site, resulting in a net loss of one carbon and two hydrogen atoms. The ten-eleven translocases (TETs) family of methylcytosine dioxygenases including TET1–3, collaborate with DNA damage 45-beta (Gadd45β), Dnmts and HDACs, to catalyze oxidation of 5mC to 5-hydroxymethylcytosine (5hmC), thus promoting DNA demethylation (reviewed in Chen and Riggs, 2011).

In post-mortem brains of patients with SZ, DNA methylation has been assayed for a number of genes mainly expressed in neurons, namely, Reelin, catechyl-O-methyltransferase (COMT), OPRM (opioid receptor, mu), the serotonin-2A receptor gene (HTR2A), brain derived neurotrophic factor (BDNF) and arachidonate 5-lipoxygenase (ALOX5; reviewed in Grayson and Guidotti, 2013). Research has reported an approximately twofold increase of SAM level in SZ (Guidotti et al., 2007). In addition, other researchers have reported that SAM levels regulate the DNA methylome of Schwann cells, which are myelination glial cells in the PNS (Varela-Rey et al., 2014). Furthermore, it was also found that there was a higher expression of Dnmt1 and Dnmt3a in SZ than patients without SZ (Guidotti et al., 2007; Zhubi et al., 2009). Besides neurons, Dnmt1, Dnmt3a and Dnmt3b are also expressed in glial cells (Feng et al., 2005). Dnmt3a-deficient NSCs tend to differentiate into astrocytes and oligodendrocytes as a result of demethylation of glial genes (Wu et al., 2012b). This data suggests a potential role of DNA methylation in glial cells which may partially explain the etiology of SZ. Moreover, evidence has shown that abnormal glial cells, such as astrocyte or oligodendrocyte dysfunction conjunction with myelin deficits occur in white matter. Interestingly, these changes are thought to be the result of their DNA methylated status changes in SZ (Iwamoto et al., 2005, 2006; Wockner et al., 2014).

### DNA Methylation in Oligodendrocytes

Studies of Dnmt3a knockout NSCs indicates that Dnmt3a may be involved in regulating fate determination of the oligodendroglial lineage (Table 1). In Dnmt3a-deficient NSCs, the methylation levels of oligodendroglial differentiation related genes such as PDGFRα, Olig1, Sox10, MBP, Id2, Id4, Nkx2.2 and Nkx6.2 are decreased, which results in up-regulation of these genes and enhanced generation of oligodendroglial cells (Wu et al., 2010, 2012b). In addition, other researchers have shown that in MeCP2 null mice the loss of MeCP2 in the oligodendrocyte lineage cells specifically resulted in more active behaviors with corresponding severe hind limb clasping phenotypes. Moreover, these MeCP2 null mice displayed reduced expression of some myelin-related proteins such as CNPase and MBP (Vora et al., 2010; Wu et al., 2012a; Nguyen et al., 2013). On the other hand, Id2/4 was shown to be demethylated during oligodendroglial differentiation, which is mediated by protein arginine N-methyltransferase 5 (PRMT5; Huang et al., 2011).

**Table 1:** **DNA methylated sate and glia related gene expression**.

Gene	Methylated state	Expression	Reference	Condition
MAG	Demethylated	Increase	Grubinska et al. (1994)	Differentiation of culture OLs
MBP	Demethylated	Increase	Wu et al. (2010)	Dnmt3a^−/−^ NSCs
	Methylated of arginine	Increase	Nguyen et al. (2013)	Development
Olig1	Demethylated	Increase	Wu et al. (2010)	Dnmt3a^−/−^ NSCs
Sox10	Hypermethylated	Decrease	Iwamoto et al. (2005); Iwamoto et al. (2006) and Wockner et al. (2014)	Schizophrenia
		Decrease	Nielsen et al. (2012)	Cocaine exposure
PAD2/4	Decreased methylation	Increase	Mastronardi et al. (2007)	Multiple sclerosis (MS)
Id2/4	Demethylated	Increase	Huang et al. (2011)	NSCs differentiation
		Increase	Wu et al. (2010)	Dnmt3a^−/−^ NSCs
GFAP	Demethylated	Increase	Takizawa et al. (2001) and Hatada et al. (2008)	Gliogenesis
	MeCP2 E1 bind to exon 1 of Gfap	—	Tsujimura et al. (2009)	NSCs
S100**β**	Demethylated	Increase	Hatada et al. (2008)	Gliogenesis
EAAT2	Hypermethylated	Decrease	Zschocke et al. (2007)	Glioma cell lines
tPA	Demethylated	Increase	Zhang et al. (2014)	Ethanol exposure
Kir4.1	Hypermethylated	Decrease	Nwaobi et al. (2014)	Ischemic, injury, epilepsy, and Alzheimer
MCT4	Hypermethylated	Decrease	Liu et al. (2014)	Temporal lobe epilepsy

*In vitro* studies involving OPC cultures have identified two Hpa2 sites located at −1836 and −39 of the MAG gene that are progressively demethylated during differentiation (Grubinska et al., 1994), thereby altering the normal myelination process. Recently, it was found that TET1-3 family members can regulate the differentiation of OPCs. Specifically, TET2 is thought to be critical for the expression of some important myelin genes, such as MBP (Zhao et al., 2014; Table 1). Furthermore, the level of 5hmC exhibited dynamic changes, indicating that the combination of DNA methylation along with demethylation may play an important role in the regulation of myelin gene expression and OPCs’ differentiation (Zhao et al., 2014).

In SZ, Sox10 hyper-methylation was found to be correlated with its reduced expression and oligodendrocyte dysfunction. However, the CpG island of Olig2 and the methylated state of MOBP, which encodes the structural protein in mature oligodendrocytes, is rarely methylated in normal brains (Iwamoto et al., 2005, 2006; Wockner et al., 2014). Recent investigations on the corpus callosum following the administration of cocaine showed a change in DNA methylation at the promoter region of the Sox10 gene. However, the methylated state of the myelin proteins such as MBP or PLP1 was not affected (Nielsen et al., 2012).

Methylation has also been shown to be involved in myelin protein integrity. For example, MBP arginine methylation level decreased following brain development, which is critical for MBP synthesis. However, in rats exposed to arsenic, MBP arginine methylation level was increased as compared to normal controls (Chanderkar et al., 1986; Zarazua et al., 2010). Moreover, methylation in the promoter of the peptidylarginine deiminase 2 (PAD2; a key enzyme which converts arginines of MBP into citrullines) was decreased to one-third of normal control in white matter of patients with multiple sclerosis (MS; Mastronardi et al., 2007). Recently, transcriptional regulation of BDNF by MeCP2 was believed as a novel mechanism for re-myelination and/or myelin repair involved in the treatment of MS (KhorshidAhmad et al., 2015).

### DNA Methylation in Astrocyte

Astrocytes are the most abundant glial cells which are known to play crucial roles in brain development (Namihira and Nakashima, 2013). Astrocytes are generated from NPCs at early-gestational to mid-gestational stages. They are located in the ventricular and subventricular zone in late gestation. During astrocytogenesis, cytokine-induced activation of the janus kinase (JAK)-signal transducer and activator of transcription (Stat) pathway are necessary. Activated Stat3 binds the promoter of Gfap gene to active gene expression, thus inducing NPCs to differentiate into astrocytes (Namihira and Nakashima, 2013). On embryonic day 11.5 (E11.5), the Stat3 binding site at the promoter region of Gfap gene is highly methylated, causing its expression silenced. However, on E14.5, the promoter of Gfap gene is demethylated thereby promoting its expression (Takizawa et al., 2001; Hatada et al., 2008). In addition to the promoter, the exon 1 of Gfap gene can also be modified by methylation. MeCP2E1, an isoform of MeCP2, binds to exon 1 of Gfap gene to suppress Gfap gene expression (Tsujimura et al., 2009). Moreover, under its ectopic expression, MeCP2 can prevent Gfap transcription and astrocytic differentiation from NSCs, even in the presence of astrocytic induced cytokine (Urayama et al., 2013).

As exposed to bromo-deoxyduridene (BrdU) or azacytidine, Gfap is demethylated and this induces astrocytic differentiation from NSCs (Schneider and d’Adda di Fagagna, 2012). Several other targets have been linked to the demethylation of the Gfap promoter. Specifically, Notch signaling, the hypoxia-inducible factor 1a (HIF1α), Old astrocyte specifically induced substance (OASIS), the murine homologs of chicken ovalbumin upstream promoter transcription factors I and II (COUP-TFI/II) are typical target molecules that contribute to the demethylation of the Gfap promoter (Naka et al., 2008; Mutoh et al., 2012; Saito et al., 2012). Interestingly, the CpG sites in Gfap gene promoter are heavily methylated in microglia, while demethylated in astrocytes (Barresi et al., 1999). Without methylation, histone modifications by H3K9me3 or H3K27me3 are not sufficient for activation of Stat3-binding site in the Gfap promoter region in NSCs. These facts indicate that DNA methylation plays a critical role in deciding the timing of astrocytogenesis (Urayama et al., 2013). In addition, the gene of S100β is also hypo-methylated during astrocytogenesis on E11.5. It has also been shown that following chemical exposure of ethanol, the protein levels of Dnmt3a and activity of Dnmts become increased, causing demethylation of the tissue plasminogen activator (tPA) promoter and resulting in increased expression in of these proteins in astrocytes (Zhang et al., 2014).

Recent research has also shown that hypo-methylation in astrocytes alters the expression of EAAT2, a family member of glutamate transporters (Zschocke et al., 2007). Interestingly, researchers have also reported that the down-regulation of MeCP2 did not affect cell morphology, growth, and cytotoxic reaction, but reduced expression of EAAT1/2 in astrocytes (Okabe et al., 2012). However, MeCP2 deficiency in astrocytes has been shown to cause significant abnormalities in BDNF regulation, cytokine production, and neuronal dendritic induction *via* non-cell-autonomous mechanism on gap junction (Maezawa et al., 2009). In SZ, the expression of EAAT2 was significantly reduced in astrocytes, suggesting that abnormal methylation status of EAAT1/2 in astrocytes may be involved in its pathogenesis (Katsel et al., 2011a).

### DNA Methylation in Glial Cells and Other Mental Disorders

Rett syndrome (RTT) is a childhood white matter disorder caused by mutations in MeCP2. It is an X-linked neuro-developmental disorder featured by autism and severe mental retardation and neurological dysfunction in females. Although RTT is generally attributed to be a primary neuronal dysfunction, it has recently been shown that astrocytes, oligodendrocytes and microglia also contribute to RTT pathophysiology (Maezawa et al., 2009; Derecki et al., 2012; Nguyen et al., 2013). MeCP2 deficiency results in oligodendrocytes and astrocytes abnormities as mentioned above. MeCP2-null microglia was shown to produce a fivefold higher level of glutamate. As such, the blockage of microglial glutamate released by gap junction and glutamate receptor antagonists attenuated the neurotoxicity associated with MeCP2-null microglia (Maezawa and Jin, 2010).

Genome-wide methylation map has identified significant changes in methylation of astrocytic markers such as Gfap, aldehyde dehydrogenase 1 family, member L1 (ALDH1L1), SOX9, glutamate-ammonia ligase (GLUL), the sodium/phosphate cotransporters NaPi-IIc (SCL34A3), gap junction alpha 1 (GJA1) and gap junction beta 6 (GJB6), brain-enriched guanylate kinase-associated protein (BEGAIN) and glutamate receptor ionotropic kainate 2 (GRIK2). These markers can serve as functional candidates involved in the clinical screening of patients for susceptibility to SZ (Nagy et al., 2015). In addition, the expression pattern of these markers has been suggested to correlate with changes in depression that is often suffered by patients with SZ. For these reasons, the significant differences in the methylation patterns specific to astrocytic dysfunction are now thought to be associated with depressive psychopathology (Nagy et al., 2015).

Kir4.1 is a glial-specific potassium (K+) channel which is essential for the development of the CNS. As such, decreased Kir4.1 expression is associated with Dnmt1-mediated DNA hyper-methylation in medical conditions such as ischemic injury, epilepsy, and Alzheimer’s disease (Nwaobi et al., 2014). In astrocytes, hypermethylation of monocarboxylate transporter 4 (MCT4) gene results in a significantly lower expression of MCT4, which subsequently leads to neuronal hyper-excitability and epileptogenesis in temporal lobe epilepsy patients (Liu et al., 2014).

## Clinical Implications and Future Directions

Based on the comprehensive review of the current literature, we have identified the importance of abnormal DNA methylation and demethylation in psychiatric disorders such as SZ. Specifically, research has identified aberrant methylation, growth arrest and DNA damage 45-beta (Gadd45β), as part of the DNA-demethylation pathway component, whose expression was increased by nearly threefold in SZ (Matrisciano et al., 2011). DNA methylation combineds with histone deacetylation regulate gene expression and represent two key focal points for epigenetic therapeutic interventional strategies for SZ. As such, evidence shows that adjunctive therapy for SZ may be more effective by combining demethylation with histone deacetylation (Dong et al., 2008, 2010). Because aberrant DNA methylation and histone deacetylation patterns are potentially irreversible, it has been suggested that therapy based on inhibiting DNA demethylation and histone deacetylation may serve as novel intervention strategies for early stage SZ. For example, when SZ-like mice being treated with antipsychotic drug clozapine and HDAC inhibitor VPA, Gadd45β expression can be activated and bind specific promoter regions of Reelin and GAD67 to cause hypomethylation, and thereby be beneficial for SZ treatment (Dong et al., 2008, 2010; Matrisciano et al., 2011).

Since glial abnormalities are thought to be key players in SZ pathology, new strategies that target the glial cells may be beneficial in the treatment of SZ (Takahashi and Sakurai, 2013; Guidotti and Grayson, 2014; Bernstein et al., 2015). Furthermore, since DNA methylation has an important role in regulating expression of glial genes DNA methylation (Table 1), studies involving more glial candidate genes should be conducted to elucidate their effects in the underlying pathogenesis of SZ. Henceforth, the advanced understanding of the specific mechanisms involving DNA methylation in regulating glial genesis and their pathological roles in SZ provide new insights into interventional treatment strategies for SZ and other related illnesses.

## Conflict of Interest Statement

The authors declare that the research was conducted in the absence of any commercial or financial relationships that could be construed as a potential conflict of interest.

## Abbreviations

Basic helix-loop-helix: bHLH
Brain derived neurotrophic factor: BDNF
Central nervous system: CNS
2,3-cyclicnucleotide-phosphodiesterase: CNP
Cytosine-guanine dinucleotide: CpG
Disrupted-in-schizophrenia 1: DISC1
DNA methytransferases: Dnmts
The excitatory amino-acid transporter: EAAT
Embryonic day 11.5: E11.5 d
Growth arrest and DNA damage 45-beta: Gadd45β
Glial fibrillary acidic protein: Gfap
Histone deacetylases: HDACs
5-hydroxymethylcytosine: 5hmC
Inhibitor of DNA binding 2 and 4: Id2/4
Myelin associated glycoprotein: MAG
Methyl-CpG-binding protein 2: MeCP2
Methyl—binding domain protein: MBD
5-Methylcytosine: 5mC
Monocarboxylate transporter 4: MCT4
Myelin oligodendrocyte glycoprotein: MOG
Myelin-associated oligodendrocytic basic protein: MOBP
Multiple sclerosis: MS
Neuroepithelial cells: NPCs
Neuregulin 1: NRG1
Neural stem cells: NSCs
Oligodendrocytes precursor cells: OPCs
Oligodendrocyte transcriptional factor 2: Olig2
Oligodendrocytes: OLs
Platelet derived growth factor receptor alpha: PDGFRα
Proteolipid protein: PLP
Peripheral nervous system: PNS
Protein arginine N-methyltransferase 5: PRMT5
S-adenosyl homocysteine: SAH
S-adenosyl-L-methionine: SAM
S100 beta: S100β
Sex-determining region Y-box containing gene 10: Sox10
Signal transducer and activator of transcription 3: Stat3
Schizophrenia: SZ
The ten-eleven translocases: TETs
The tissue plasminogen activator: tPA.
